# The relationship between the use of social networks and symptoms of social anxiety

**DOI:** 10.1192/j.eurpsy.2023.456

**Published:** 2023-07-19

**Authors:** F. Z. Chamsi, E. A. Adil

**Affiliations:** ^1^psychiatry department, CHU tanger, tanger; ^2^psychiatry department, university hospital tangier morocco, tanger, Morocco

## Abstract

**Introduction:**

Facebook Instagram, tik tok are popular platforms for interacting with others to build or maintain relationships. Compared to other interpersonal exchanges, these social networks do not require face-to-face interactions. Therefore, they may represent an important social sphere for people with social anxiety disorder (SAD). This study investigated the relationship between social anxiety symptoms and different patterns of social media use. We also looked at the role of brooding, a known risk factor for Social Anxiety Disorder.

**Objectives:**

establish the relationship between the misuse of social media and the symptoms of social anxiety

**Methods:**

This is an analytical cross-sectional study conducted through the distribution of a self-questionnaire, which was published online, through different platforms. The questionnaire in the 1st part includes socio-demographic data and general questions about social networks. And a Social Phobia Scale (SPS) which has 20 items in a second part.

**Results:**

preliminary results showed that greater social anxiety symptoms were associated with spending more time on social media and using passively (i.e. viewing other people’s profiles without interacting). .

Social anxiety is a fear associated with certain social activities or performance situations where the person might feel observed, embarrassed, humiliated, rejected or concerned about the judgment of others.

In people with social anxiety, however, these fears become excessive, persistent and pervasive. The affected person may panic and attempt to avoid situations or conditions that remind them of the object of their fear. While the use of social networks especially among adolescents can create a virtual space in which users can act and react freely without coming into direct contact with people, which increases the signs of social anxiety in this type of user.

**Conclusions:**

the relationship between social media use and social anxiety symptoms highlights the need for further research on this topic. Future research should use experimental manipulations and replicate current findings in clinical samples.

**Disclosure of Interest:**

None Declared

